# Increases in total HIV-1 nucleic acid in whole blood precede plasma RNA rebound during pediatric analytical treatment interruption

**DOI:** 10.1097/QAD.0000000000004506

**Published:** 2026-04-24

**Authors:** Gabriela Z.L. Cromhout, Nomonde Bengu, Nicholas G. Herbert, Rowena Fillis, Samantha Kannie, Jeroen Van Lobenstein, Kogielambal Chinniah, Malini Krishna, Roopesh Bhoola, Noxolo Mahlaba, Maria C. Garcia-Guerrero, Thumbi Ndung’u, Maria C. Puertas, Javier Martinez-Picado, Moherndran Archary, Philip J.R. Goulder

**Affiliations:** a HIV Pathogenesis Programme, Doris Duke Medical Research Institute, Nelson R Mandela School of Medicine; b Department of Paediatrics and Child Health, University of KwaZulu-Natal, Durban; c Queen Nandi Regional Hospital, Empangeni, South Africa; d Peter Medawar Building for Pathogen Research, Department of Paediatrics, University of Oxford, Oxford, UK; e Harry Gwala Regional Hospital, Pietermaritzburg; f General Justice Gizenga Mpanza Regional Hospital, KwaDukuza; g Mahatma Gandhi Memorial Hospital; h School of Laboratory Medicine and Medical Sciences College of Health Sciences University of KwaZulu-Natal, Durban, South Africa; i Ragon Institute of Massachusetts General Hospital, Massachusetts Institute of Technology and Harvard University, Cambridge, Massachusetts, USA; j Africa Health Research Institute, Durban, South Africa; k Division of Infection and Immunity, University College London, London, UK; l IrsiCaixa, Badalona, Barcelona; m Institute for Health Science Research Germans Trias i Pujol (IGTP), Badalona; n Consorcio Centro de Investigación Biomédica en Red de Enfermedades Infecciosas (CIBERINFEC), Instituto de Salud Carlos III, Madrid; o University of Vic-Central University of Catalonia (UVic-UCC), Vic; p Catalan Institution for Research and Advanced Studies (ICREA), Barcelona, Spain.

**Keywords:** Africa, analytical treatment interruption, antiretroviral therapy, cure/remission, HIV, HIV-1, HIV replication, pediatrics, point-of-care testing

## Abstract

**Objective(s):**

In the absence of reliable biomarkers, analytical treatment interruption (ATI) is needed to evaluate HIV-1 cure/remission strategies. Children living with HIV-1 may have high potential to achieve cure/remission due to very-early antiretroviral therapy (ART) initiation combined with early-life immunity. However, pediatric ATI safety monitoring requires frequent blood sampling for early plasma viral rebound detection to facilitate prompt ART resumption.

**Design:**

We hypothesized that viral rebound during ATI would be detected earlier by point-of-care (PoC) total HIV-1 nucleic acid (TNA) measurement, which quantifies both HIV-1 RNA and DNA, than via standard laboratory assays of plasma HIV-1 RNA. Therefore, viral rebound during pediatric ATI studies could be detected earlier via a practical and user-friendly approach.

**Methods:**

Nineteen very-early ART-treated children underwent ATI. Sixteen experienced plasma HIV-1 viral rebound. PoC TNA testing with the Cepheid GeneXpert HIV-1 Qual XC assay, requiring 100 μl of whole blood and 2 h turnaround time (TAT), was compared with standard plasma HIV-1 RNA quantification (Aptima Quant), requiring 500 μl of whole blood and 30 h TAT.

**Results:**

GeneXpert TNA Ct values and plasma HIV-1 RNA were strongly negatively correlated (*r* = -0.88, *P* < 0.0001). In all 27 instances where viral rebound was detected by plasma HIV-1 RNA testing, TNA was also detected. In 12 instances, TNA was detected prior to any plasma RNA rebound. There were no instances where plasma rebound was detected prior to TNA detection (*P* = 2 x 10^–15^).

**Conclusion:**

PoC HIV-1 TNA testing offers a more rapid and lower-volume method to detect viral rebound in pediatric ATI, enhancing safety monitoring and timely ART reinitiation.

## Introduction

An estimated 1.4 million children are living with HIV (CLWH) [1,2]. Although antiretroviral therapy (ART) is available, only 55% of CLWH are on ART and, of these, only 47% are virally suppressed [1]. Progression to late-stage HIV disease and death is especially rapid in children in the absence of ART, with a 60% 2-year mortality [3]. While early ART initiation improves survival, alternatives to life-long ART alone are urgently needed for CLWH. Paradoxically, if ART can be initiated very early, the potential for HIV-1 cure/remission may be higher in children than adults, because of the tolerogenic impact of early-life immunity [3–7]. This has led to the necessity to develop strategies to facilitate HIV-1 cure/remission in CLWH.

Cases of posttreatment control/ART-free remission in children have included the Mississippi Baby [8], the French Adolescent [9], the South African Child [10], and, more recently, five boys from the Ucwaningo Lwabantwana Cohort [11] and four children within the IMPAACT P1115 trial [12]. However, apart from the IMPAACT P1115 trial, these studies have all depended upon unscheduled ART interruptions to identify those children capable of maintaining ART-free control of viremia. Prospective ATI trials provide the only opportunity to confirm and document the precise duration of ART-free viral control and to obtain the clinical samples from which the underlying mechanisms of cure/remission can be determined. Consequently, a number of prospective ATI studies in children are ongoing or in development, including IMPAACT P1115 [12–14], which identified four children who achieved ART-free viral suppression for more than 12 months, IMPAACT 2042/Tatelo-Plus [15,16], IMPAACT P2039 [17] and our own study, Azaphile [18], the ATI substudy nested within the Ucwaningo Lwabantwana cohort.

In order to ensure well tolerated interruption of ART, robust monitoring during ATI is essential and typically includes frequent measurement of plasma HIV-1 RNA using both laboratory-based and, where feasible, point-of-care (PoC) assays. However, PoC plasma viral load testing requires additional infrastructure at clinic sites for plasma separation, requires 500–1000 μl of plasma [19–22] and has higher lower limits of quantification (LLoQ) than standard laboratory assays: for example, the Aptima Quant laboratory assay has a lower limit of quantification (LLoQ) of 30 HIV-1 RNA copies/ml plasma (requiring 500 μl of plasma) [23], whereas the Alere PoC has a LLoQ of 1000 c/ml [20]. Moreover, in pediatric studies, to limit the blood volumes sampled, plasma is often diluted in testing [24], compromising assay sensitivity even further.

We hypothesized that PoC testing for total nucleic acid (TNA) might provide a more effective alternative to standard, laboratory-based plasma viral load assays, TNA measurement includes HIV-1 RNA and DNA in whole blood in early-treated CLWH. To test this hypothesis, we monitored 19 children participating in our Azaphile ATI study [18], using both the Cepheid whole blood PoC TNA assay and a standard laboratory-based assay (Aptima Quant, Hologic, San Diego, CA, USA), which has a LLoQ of 30 c/ml, to measure plasma HIV-1 viral load.

In addition to evaluating TNA as a rebound detection tool during ATI, we examined the relationship between PoC TNA Ct values, plasma HIV-1 RNA viral loads, and total HIV-1 DNA across the broader Ucwaningo Lwabantwana Cohort (ULC), in order to contextualize the clinical positioning and generalizability of PoC TNA testing in pediatric HIV monitoring.

## Materials and methods

This study is nested within the Ucwaningo Lwabantwana Cohort (ULC) [11,25,26], a cohort based in KwaZulu-Natal, South Africa. From this larger cohort, a subset of participants (*n* = 19) underwent a time-to-rebound ATI. A time-to-rebound ATI is defined here as an ATI wherein ART is discontinued under strict supervision and monitoring, and then resumed following confirmation of any detectable plasma HIV-1 RNA rebound (>30 HIV-1 RNA c/ml) on standard laboratory testing.

### Participants

The ULC is a longitudinal cohort of more than 325 mother--child pairs where the children acquired HIV *in utero*, were started on ART within 21 days of life and received standard-of-care (SoC) ART, as per South African Department of Health guidelines. Participants were based across four clinical sites in KwaZulu-Natal, South Africa. The ATI was undertaken in children enrolled on the Azaphile trial [PACTR202412878025175] [18]. These children were older than 3 years old, had maintained undetectable (<30 c/ml) plasma viral loads for 24 months or longer on standard laboratory assays, had CD4^+^ cell counts normal for age (as per WHO guidelines) [27] and had all measures of HIV DNA undetectable/less than 20 c/million PBMC and/or TNA HIV-1 GeneXpert undetectable/with a Ct more than 40 in the preceding 12 months, as well as giving informed consent and assent. In addition to this, participants were excluded from the ATI if they had any evidence of failure to thrive, had confirmed/suspected tuberculosis (TB), were receiving TB preventive therapy or had any medical condition that would make participation in the study unsafe, complicate interpretation of study outcome data, or interfere with achieving the study objectives. All met the standard nucleic acid criteria for HIV-1 infection. This entailed, at birth, all infants undergoing SoC dried blood spot TNA PCR testing (COBAS AmpliPrep/COBAS TaqMan HIV-1 Qualitative PCR v2, Roche Diagnostics) performed at a central laboratory. Positive or indeterminate results were confirmed or repeated at approximately 7 days of age. Infants born to mothers living with HIV received ART within minutes of delivery (nevirapine [NVP] alone or zidovudine [AZT] plus NVP, per South African guidelines). Those at high risk of in utero HIV transmission additionally underwent PoC whole-blood TNA PCR testing (GeneXpert HIV-1 Qualitative, Cepheid, CA, USA) immediately after birth.

### Procedures

**Fig. 1:**
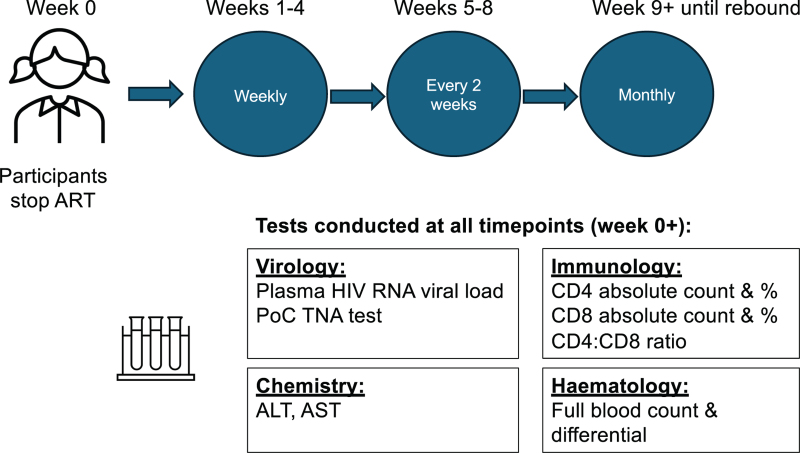
Safety monitoring schedule during analytical treatment interruption.

Participants enrolled on the ATI underwent rigorous safety monitoring, which included thorough history, clinical examination, laboratory-measured plasma HIV-1 viral load, PoC TNA testing, immunological monitoring (including absolute CD4^+^ level, CD4%, CD4:CD8 ratio), hematological (specifically full blood count with differential blood count) and chemistry (including ALT and AST monitoring).). This monitoring took place weekly for the first month during ATI, then fortnightly for a second month and then monthly (Fig. 1). Interim visits were conducted as deemed necessary based on caregiver/participant history between these visits. HIV-1 TNA was measured via PoC testing using the Cepheid GeneXpert Qual XC assay (GXP), which requires 100 μl of whole blood per test. Plasma HIV-1 RNA was measured utilizing standard laboratory testing using the Hologic Aptima HIV-1 Quantitative assay which requires 1 ml whole blood for the 500 μl of plasma needed for the test. Stored plasma and peripheral blood mononuclear cells (PBMCs) were utilized for confirmatory testing and quantification of total HIV-1 DNA by droplet digital PCR (ddPCR; BioRad, CA, USA) [11].

### Ethics

This study was ethically approved by the University of KwaZulu-Natal's Biomedical Research Ethics Committee (BREC) and overseen by a Data and Safety Monitoring Board (DSMB). Written informed consent and assent (where age and maturity-appropriate) were obtained from the participants and their legal guardians in their language of preference. Permitted blood volume maximums taken on each child were in keeping with the Stellenbosch University Health Research Ethics Committee's guidelines of weight-based volumes.

### Statistical analysis and methods

Baseline characteristics and laboratory results were described utilizing absolute numbers, percentages, median, and interquartile ranges (Table 1). The relationship between the plasma RNA viral load and PoC TNA Ct, Total HIV-1 DNA and PoC TNA Ct was evaluated through the use of Spearman's nonparametric correlation. Comparisons were performed using the Chi-squared and Fisher's exact tests for categorical variables and the Mann-Whitney *U*-test for continuous variables. *P* values of less than 0.05 were considered to be statistically significant. Sample size was determined according to the number of participants fulfilling the eligibility criteria, as they make up a unique subset of the population and, therefore, power calculations were not conducted. Analyses and calculations were conducted using Graphpad Prism Version 10.5.0. Although a formal sensitivity analysis was not performed, diagnostic accuracy estimates were interpreted in the context of known limitations, such as variability in test performance across clinical subgroups.

**Table 1:** Demographic and clinical information of participants at baseline.

Characteristic	Median	IQR	*n* (%)
Demographic characteristics			
Age (months)	60	49–77	–
Sex			
Male	–	–	7 (44)
Female	–	–	9 (56)
Clinical characteristics			
Vertical transmission prophylaxis (1–2 drug ART) initiation (day of life)	0	0–0	–
Maternal ART regimen prior to delivery			
FTC/TDF/EFV (TEE)	–	–	12 (75)
TDF/3TC/DTG (TLD)	–	–	1 (6)
FTC/TDF/Lpv/r	–	–	1 (6)
Nil (ART naive at delivery)	–	–	2 (13)
Triple therapy ART initiation (days)	9	3–14	–
Baseline absolute CD4^+^ cell count (cells/μl)	2805	2139–3198	–
Baseline CD4%	51	31–57	—
Baseline CD4:CD8 ratio	2.30	1.53–2.57	–
Baseline plasma viral load (copies/mL)	985	<30–20 425	–
Baseline total HIV DNA load (copies/10^6^ PBMCs)	148	23–398	–
Age at viral suppression [pVL <30 copies/ml] (months)	1.5	0–2.8	–
Duration of viral suppression (months)	54	45–73	–
Pre-ATI total HIV DNA load (copies/10^6^ PBMCs)	2	0–4	–
Pre-ATI HIV-1 total nucleic acid (TNA) Ct	Not detected	0–43.1	–

3TC, lamivudine; ART, antiretroviral therapy; Ct, cycle threshold; DTG, dolutegravir; EFV, efavirenz; FTC, emtricitabine; Lpv/r, lopinavir/ritonavir; PBMCs, peripheral blood mononuclear cells; pVL, plasma viral load; TDF, tenofovir.

In addition to the ATI-specific analyses, we evaluated the broader relationship between PoC TNA Ct values, plasma HIV-1 RNA viral loads, and total HIV-1 DNA across the wider ULC cohort. This included assessment of TNA and total HIV-1 DNA detectability in ART-treated children with undetectable plasma viral loads for more than 24 months (*n* = 708); correlation between PoC TNA Ct and plasma HIV-1 RNA in all children (*n* = 1096 paired comparisons); correlation between PoC TNA Ct and plasma HIV-1 RNA restricted to viremic children; and correlation between PoC TNA Ct and total HIV-1 DNA load. Spearman's nonparametric correlation was used for all correlation analyses.

## Results

### Study participants

Between October 2022 and June 2025, 16 participants (*n* = 16) from the ULC were eligible for, consented to, and underwent ATI and experienced plasma HIV-1 viral rebound during the ATI period. The baseline demographics and clinical characteristics are shown in Table 1. In total, 19 participants from the ULC were eligible for and consented to ATI [30]. Of these, 16 experienced plasma HIV-1 viral rebound during the ATI monitoring period and form the basis of the diagnostic comparison presented here.

### HIV-1 plasma viral rebound during analytical treatment interruption

**Fig. 2:**
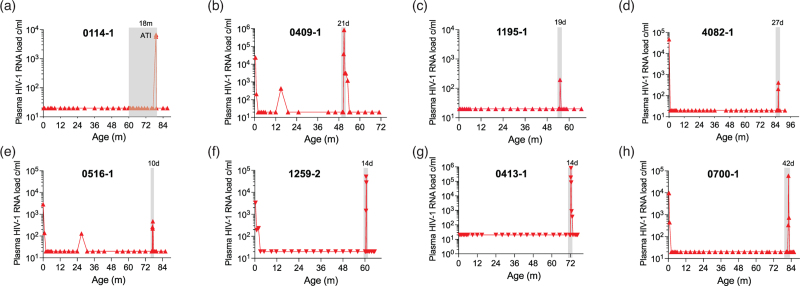
Plasma HIV-1 RNA viral loads from birth through to the ATI for 8 representative participants of pediatric ATI study: (a) 0114-1. (b) 0409-1. (c) 1195-1. (d) 4082-1. (e) 0516-1. (f) 1259-2. (g) 0413-1. (h) 0700-1. Remainder of participant data are shown in supplementary Fig 1.

**Fig. 3:**
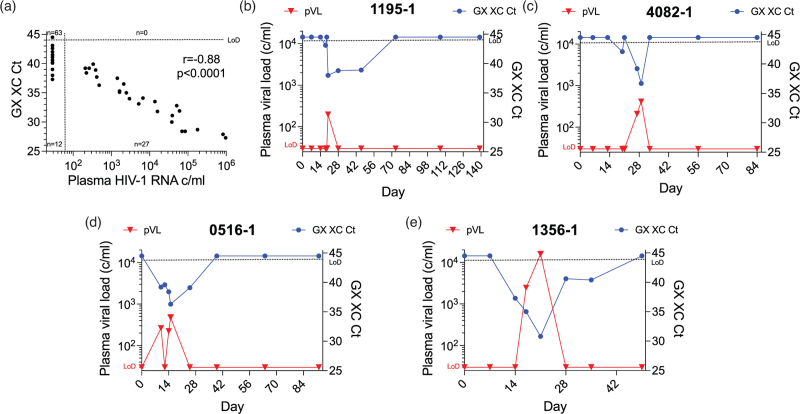
(a) Correlation between HIV-1 pVL and the PoC TNA Ct values data from all timepoints (*n* = 102) during the ATI up to the time of viral rebound for *n* = 16 (*r* = -0.88, *P* < 0.0001). B-E. HIV-1 plasma viral loads and PoC TNA Ct values for 4 representative participants: (b) 1195-1. (c) 4082-1. (d) 0516-1. (e) 1356-1.

Plasma HIV-1 RNA viral loads from birth through to the ATI for the 16 study participants are shown in Fig. 2 and Supplementary Figure 1. Viral rebound was defined as a confirmed plasma HIV-1 RNA viral load (pVL) of more than 30 c/ml. Analyzing all timepoints (*n* = 102) during the ATI up to the time of viral rebound for all 16 participants, we observed a strong negative correlation between HIV-1 pVL and the PoC TNA Ct values (*r* = -0.88, *P* < 0.0001, Fig. 3a). HIV-1 plasma viral loads and the PoC TNA Ct values are shown for four representative participants (Fig. 3b--e). These data show that, in five of these 16 participants undergoing ATI, HIV-1 plasma viral rebound was preceded by TNA rebound, and in the remaining participants, HIV-1 pVL rebound and TNA rebound were contemporaneous. There were no instances of HIV-1 pVL rebound preceding TNA rebound. The TNA PoC test therefore detects viral rebound before HIV-1 pVL becomes detectable in more than 30% of the participants studied here. Taking into account the practical advantages of a PoC test, the TNA PoC test would represent a valuable part of safety monitoring in pediatric ATI trials such as the one described here. In the five participants (31%) in whom TNA detection preceded plasma RNA rebound, this occurred one to two scheduled visits earlier. Because the PoC TNA result was available within 2 h at the clinic, detection of TNA at a scheduled visit triggered additional follow-up visits, enabling repeat testing and earlier confirmation of viral rebound than would have been possible with standard laboratory testing alone. In the remaining 11 participants, TNA and plasma RNA rebound were detected contemporaneously.

### GX TNA Ct, plasma HIV-1 RNA viral load, and total HIV DNA load

**Fig. 4:**
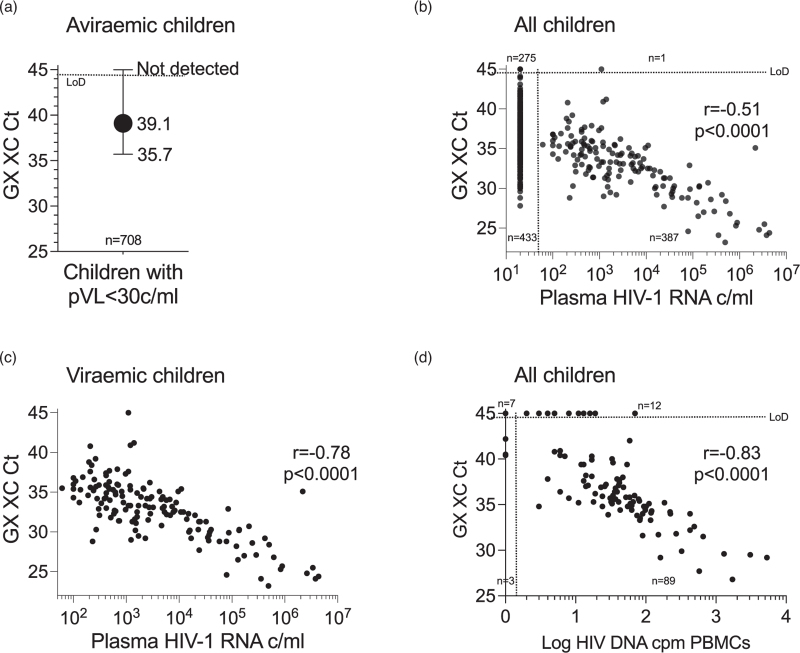
(a) Most antiretroviral therapy treated children who have undetectable plasma HIV-1 RNA viral loads for >24m do not have undetectable TNA or total HIV-1 DNA loads (*n* = 708). (b) Correlation between HIV-1 pVL and the PoC TNA Ct in all children (*r* = -0.32, *P* < 0.0001). (c) Correlation between HIV-1 pVL and the PoC TNA Ct value in viremic children (*r* = -0.78, *P* < 0.0001). (d) Correlation between HIV-1 TNA and total HIV-1 DNA load (*r* = -0.83, *P* < 0.0001).

In the Azaphile trial [18], the eligibility criteria for study participants included [1] undetectable plasma HIV-1 RNA viral loads for more than 24 months and [2] very low or undetectable total HIV-1 DNA loads and/or TNA on ART. However, most ART-treated children who have undetectable plasma HIV-1 RNA viral loads for more than 24 months do not have undetectable TNA or total HIV-1 DNA loads (Fig. 4a). As a result, the correlation between HIV-1 pVL and the PoC TNA Ct values is considerably weaker if one includes children who are virally suppressed on ART (*r* = -0.51, *P* < 0.0001, Fig. 4b). Nonetheless, as with the children whose viral rebound was evaluated in the ATI study, among the total cohort of CLWH, we again observed very few instances of a child with detectable pVL who had undetectable HIV-1 TNA (one in 1096 comparisons evaluated), whereas the reverse was relatively frequent (433 instances out of 1096 comparisons evaluated). Among all viremic children in the cohort, most of whom would have detectable HIV-1 TNA when virally suppressed, the negative correlation between pVL and HIV-1 TNA is relatively strong (*r* = -0.78, *P* < 0.0001, Fig. 4c). Directly comparing HIV-1 TNA versus total HIV-1 DNA load, these were strongly correlated, irrespective of pVL (*r* = -0.83, *P* < 0.0001, Fig. 4d).

## Discussion

In this study of very-early ART-treated children undergoing ATI, plasma viral rebound observed via standard laboratory testing was preempted in five of 16 cases by detection of PoC TNA. There was no instance in which plasma viral rebound arose without PoC TNA being detected either at the same time or at a previous timepoint.

The higher sensitivity of the PoC TNA assay in detecting viral rebound is especially valuable in pediatric ATI studies for several reasons. First, early detection of viral rebound allows ART to be resumed without delay. In time-to-rebound ATI studies, there is no benefit to prolonging the ATI; on the other hand, early ART resumption decreases the likelihood of acute retroviral syndrome (ARS), which was a feature of the P1115 study [12,13], and other unwanted consequences of high viraemia.

Second, the PoC TNA test is highly practical, in providing an immediate result (turnaround time of under 2 h) in the clinic, compared to a median turnaround time of 30 h (IQR 26- 32) for plasma HIV-1 RNA load from standard laboratory testing. The short waiting time for results also has the distinct advantage of lowering the anxiety associated with awaiting results for the study participants, their caregivers and study teams. The timing also decreases the economic strain on these participants and their caregivers. They will be required to make multiple trips for confirmatory testing and reinitiation (if appropriate) if relying solely on standard testing, but with the POC TNA testing, they require less study-related absenteeism from school and work.

Third, only 100 μl of whole blood is required for the assay, contrasting with 0.5–1.0 ml for standard laboratory quantification of plasma HIV-1 RNA. PoC plasma HIV-1 RNA load quantification requires additional infrastructure at clinic sites for plasma separation, plasma volumes of 500–1000 μl, and the LLoQ is higher than that of the standard laboratory testing (such as the Aptima Quant assay) which is 30 c/ml. In summary, especially in harder-to-reach areas in sub-Saharan Africa, where the majority of ATI studies of CLWH are likely to be conducted, PoC GX TNA testing is superior to the other assays available and will increase the safety of ATI studies in children.

It is important to note that the majority of ART-treated children with prolonged plasma viral suppression (>24 months) retain detectable PoC TNA and/or total HIV-1 DNA (Fig. 4a). This finding reflects the well established persistence of cell-associated HIV nucleic acids – including integrated proviral DNA and cell-associated RNA – in individuals on suppressive ART, rather than indicating incomplete viral suppression. Consequently, a detectable TNA result in a virally suppressed child on ART should be expected and does not indicate treatment failure.

The PoC TNA Ct values correlate most strongly with plasma HIV-1 RNA in the setting of active viraemia (*r* = -0.78, *P* < 0.0001, Fig. 4c), whereas the weaker correlation observed in the full cohort, including virally suppressed children (*r* = -0.32, Fig. 4b), reflects the contribution of cell-associated nucleic acids to the TNA signal. Accordingly, PoC TNA Ct values should not be interpreted as quantitative surrogates for plasma viral load in children with undetectable or low-level viraemia, where biological compartmentalization and assay sensitivity limits dominate. These data underscore the optimal clinical application of PoC TNA in the ATI setting, where its primary role is the sensitive detection of viral rebound rather than quantification of viral load during suppression.

A further value of the PoC GX TNA assay is the observation of a strong correlation with total HIV-1 DNA. A low total HIV-1 DNA is associated with increased potential to achieve HIV-1 cure/remission [28–30]. Thus, a PoC test that can rapidly identify children early in life who might benefit from interventions designed to facilitate HIV-1 cure/remission is valuable. This is of particular importance, since determinations of HIV-1 DNA load can be challenging and time-consuming for investigators collecting samples from CLWH in clinics within sub-Saharan Africa.

The strong correlation between PoC TNA Ct values and total HIV-1 DNA (*r* = -0.83, *P* < 0.0001, Fig. 4d) is consistent with PoC TNA serving as a practical proxy for HIV reservoir size. Since TNA encompasses both RNA and DNA, it captures overlapping but nonidentical biological compartments compared to total HIV-1 DNA alone, with TNA additionally reflecting transcriptionally active proviruses. Further studies are needed to determine the extent to which PoC TNA reflects reservoir size versus transcriptional activity, although the strong correlation observed here supports its utility as a rapid, PoC surrogate for reservoir burden in early-treated children.

The longitudinal viral load trajectories from birth through ATI (Fig. 2 and Supplementary Figure 1) illustrate the variable history of viraemia across participants. Historical viraemia and duration of suppression prior to ATI may influence baseline TNA and total HIV-1 DNA levels at the time of treatment interruption, and this is relevant to interpreting individual variation in the timing of TNA detection relative to plasma rebound.

Based on these data, we propose that PoC TNA is best positioned as follows: in the context of ATI studies and other settings where viral rebound detection is the primary objective, PoC TNA serves as a rapid, practical, and sensitive first-line detection tool. In viremic children more broadly, it functions as a complementary marker alongside plasma viral load. In ART-suppressed individuals, the data support its potential role as an exploratory surrogate marker for reservoir-associated nucleic acids, although its utility in this context requires further validation.

It is important to highlight the limitations of this study. We did not perform a formal sensitivity analysis; however, the consistency of results across multiple subgroups supports the robustness of our findings. The children eligible for this ATI study were required to have very low or undetectable levels of HIV-1 TNA and/or total HIV-1 DNA. In this group, the pVL and HIV-1 TNA Ct values were strongly correlated (*r* = -0.88, *P* < 0.0001). However, in children who are virally suppressed, only a minority have undetectable HIV-1 TNA or total HIV-1 DNA loads (Fig. 2a). Nonetheless, more than 1000 comparisons of GX TNA Ct and pVL in the ULC children support the findings from the ATI trial that GX TNA Ct and pVL are strongly correlated (Fig. 3b,c), and these data are consistent with the observation that changes in HIV-1 TNA precede plasma HIV-1 RNA rebound in children previously suppressed on ART.

A further consideration is that while the GeneXpert HIV-1 Qual XC assay provides Ct values in the research setting, in routine clinical practice, the output is typically reported as a qualitative Detected/Not Detected result, and Ct values may not be routinely available to or interpretable by clinicians. Importantly, the core findings of this study are robust to a purely qualitative interpretation: the ability to identify ATI candidates on the basis of an undetectable PoC TNA result prior to ATI remains valid, and the detection of viral rebound via a change from “Not Detected” to “Detected” would function effectively as an early safety signal during ATI monitoring. The semi-quantitative Ct data presented in Figs. 3a and 4b–d should be interpreted with this practical limitation in mind.

Regarding the generalizability of these findings, the ability of PoC TNA to detect HIV nucleic acids in the absence of detectable plasma RNA makes it potentially informative for safety monitoring in ATI and cure-directed studies across different pediatric and adult populations. However, the current study population comprises a unique group of very-early ART-treated children with exceptionally low reservoirs, and the performance of PoC TNA may differ in populations with higher baseline reservoir levels or longer durations of infection prior to ART initiation. Validation in other ATI cohorts would be valuable.

An important practical consideration is the clinical action to be taken when PoC TNA is detected during ATI. In the Azaphile study protocol, a newly detected PoC TNA result triggers expedited confirmatory plasma viral load testing and closer clinical follow-up, rather than immediate ART resumption. Because the turnaround time for PoC TNA is under 2 h (compared to ~30 h for laboratory plasma viral load), the clinical team can be alerted to the likelihood of imminent rebound in real time, allowing planning for prompt ART reinitiation once plasma viraemia is confirmed. Future ATI study designs may consider using PoC TNA detection as a direct trigger for ART reinitiation, which would further shorten the window of viremia, although this approach would need to be balanced against the possibility of restarting ART in the absence of confirmed plasma viraemia.

In summary, these data indicate that PoC GX TNA testing on whole blood contributes to safety monitoring in pediatric ATI studies, as a biomarker of imminent plasma HIV-1 RNA rebound. Including PoC TNA testing as part of safety monitoring during ATI would enable clinicians to be alerted early to the possibility of ARS and the potential need to reinitiate ART, depending on the study design. At the same time, the low volume of blood sample required for the TNA assay allows for the frequent screening needed during paediatric ATI studies without compromising on robustness.

## Acknowledgements

G.C., N.B., M.A., and P.G. wrote the study, contributed to the study conception and design, and contributed to the acquisition, analysis, and interpretation of the data; M.A., T.N., M.P., and J.M.P. contributed to the study design, and to the analysis and interpretation of the data. N.H., R.F., K.C., R.B., M.K., N.M., J.V.L., and S.K. contributed to the study design, and to the acquisition, analysis, and interpretation of the data; All authors contributed to the intellectual content, interpretation of data, critical revisions to the drafts of the study and approved the final version of the manuscript.

The data that support the findings of this study are available from the corresponding author upon reasonable request.

This work received funding from the Wellcome Trust (PG WTIA Grant WT104748MA) and the National Institutes of Health (AI133673 and U01AI168655 to P.G.; 1UM1AI164566 (the Pediatric Adolescent Virus Elimination (PAVE) Martin Delaney Collaboratory Grant) to P.G. and P.P.; AI184094, AI176579, AI155233 and AI152979 (all to M.L.); 1UM1AI164561–01 to J.M.-P.; UM1AI106716 and UM1AI068632 to E.C.; and eRA grant BX004547, VA Health Administration, Biomedical Laboratory R&D (to J.C.K.)). This study was also supported by the EPIICAL project (https://www.epiical.org) to N.C., P.P., and P.G., supported by PENTA-ID foundation (https://penta-id.org/) and funded through an independent grant by ViiV Healthcare UK. The “Ucwaningo Lwamawele” study was also supported by the grant number UO1AI168655 to P.G. Cepheid provided assistance to N.B. to help meet the costs of PoC testing and cartridges for initial testing of the ULC prior to ATI study. Cepheid also provided loan of the Xpert HIV-1 Qual XC RUO system for the duration of the study. The funders played no role in either the design or execution of the study.

### Conflicts of interest

There are no conflicts of interest.

## Supplementary Material

Supplemental Digital Content
