# Supplementary figures and images for: Increases in total HIV-1 nucleic acid in whole blood precede plasma RNA rebound during pediatric analytical treatment interruption
Source: AIDS. 2026 Apr 24;40(8):1124–32. doi: 10.1097/QAD.0000000000004506 (PMC13336579; doi:10.1097/QAD.0000000000004506)

# Supplementary Figure 1

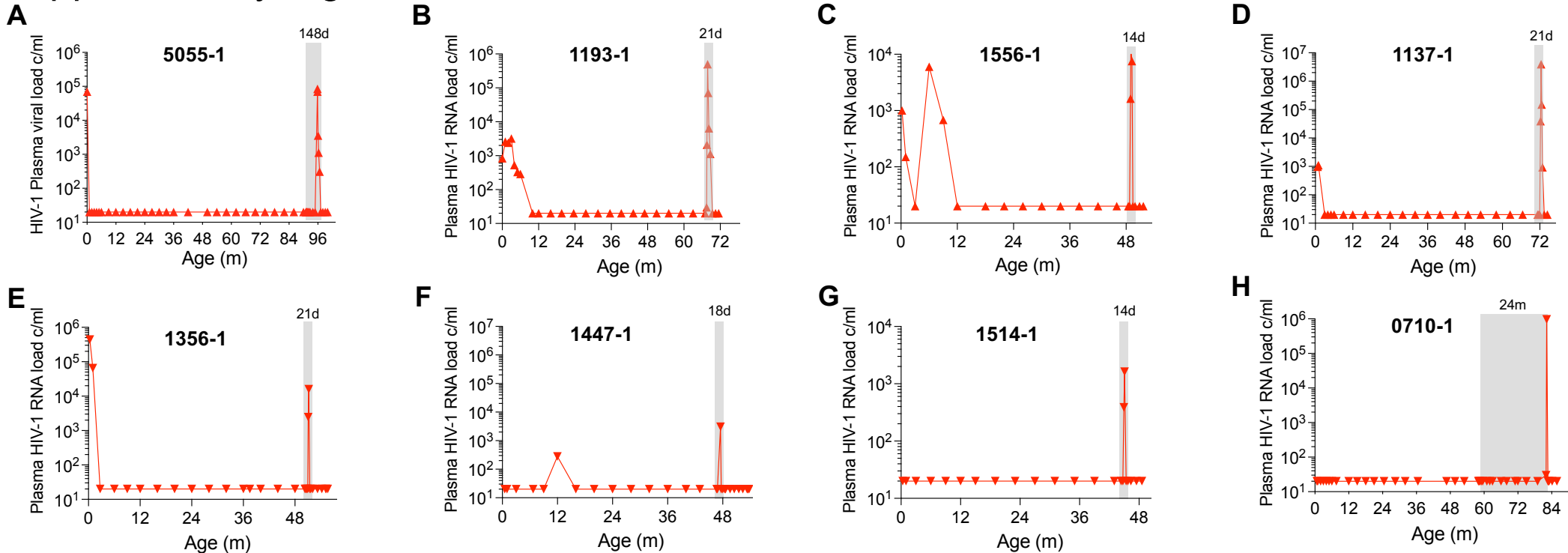

Supplement: Supplemental Digital Content [file aids-40-1124-s001.pdf]
